# Risk Factors for Postoperative Respiratory Mortality and Morbidity in Patients Undergoing Coronary Artery Bypass Grafting

**DOI:** 10.5812/aapm.5228

**Published:** 2012-09-13

**Authors:** Samira Rajaei, Ali Dabbagh

**Affiliations:** 1 Department of lab Sciences, School of Allied Medicine, Iran University of Medical Sciences, Tehran, IR Iran; 2 Anesthesiology Research Center, Shahid Beheshti University of Medical Sciences, Tehran, IR Iran

**Keywords:** Risk Factors, Postoperative Period, Mortality, Morbidity, Coronary Artery Bypass

## Abstract

**ABSTRACT:**

Nowadays, coronary artery bypass grafting (CABG) is considered to be one of the most common surgical procedures. This procedure has been the main topic in many clinical research studies, which have assessed the effect of the procedure on patients’ outcomes. Like other surgical procedures, this procedure is also accompanied by a number of unwanted complications, including those of the respiratory system. Since the respiratory system plays an integral role in defining the clinical outcome of patients, improvements in studies that can assess and predict clinical outcomes of the respiratory system, assume greater importance. There are a number of predictive models which can assess patients in the preoperative period and introduce a number of risk factors, which could be considered as prognostic factors for patients undergoing CABG. The respiratory system is among the clinical systems that are assessed in many prediction scoring systems. This review assesses the main studies which have evaluated the possible risk factors for postoperative respiratory mortality and morbidity, in patients undergoing CABG.

## 1. Introduction

Coronary artery bypass grafting (CABG) is currently a very common procedure. This major surgical procedure does, however, have a great impact on the majority of body organs. In such patients, it would be useful to determine the perioperative risk factors, which can predict the possibilities for pulmonary morbidity and mortality after cardiac surgery. Risk factor identification and assessment would improve the quality of clinical management of these patients much more accurately (1, 2). The optimization of clinical conditions before the start of the surgical procedure might help to enhance the clinical outcomes for these patients (3, 4), and could also help clinicians in their clinical management, thus preventing untoward postoperative events and complications. In addition, cardiovascular surgical teams have to be aware of potential risk factors predisposing patients to unacceptable postoperative outcomes (5).

In CABG patients, there are a number of adverse stress responses imposed on them (6) due to the nature of the procedure; including the anesthesia and surgical procedures, superimposed by the stress of the cardiopulmonary bypass (CPB) surgery, itself. These unwanted factors affect all physiologic parts of the body, including the pulmonary endothelial beds in diseased or even normal pulmonary beds, and this leads to a number of side effects on the lungs (7). A number of these changes would normally be created after all types of anesthesia, when accompanied with muscle relaxation and mechanical ventilation (8). However, there are a number of effects that are mainly seen after CABG, such as; decreased vital capacity, decreased functional residual capacity, arterial hypoxemia, and reduced lung compliance with resultant breathing work, which could increase the total oxygen utilization by up to 20% in spontaneous ventilation. Finally, it would increase the burden imposed on the myocardium, while the myocardium is ischemic (9).

One of the most important organs affected in CABG patients, is the respiratory system. Moreover, pulmonary complications are among the main complications encountered after cardiac surgery (10), and there are a number of risk factors attributed to prolonged mechanical ventilation, although not all of them are definitive. CABG patients are usually extubated within a maximum of three days after CABG. Nevertheless, there are a number of preoperative risk factors that are predictive for the occurrence of postoperative respiratory complications, and hence could induce postoperative mortality and morbidity due to respiratory problems (11, 12).

## 2. Preexisting Chronic Obstructive Pulmonary Disease

One of the main factors that can predict a patient’s risk factors for postoperative respiratory complications is the underlying lung status (1, 2). Therefore, patients with a preoperative forced expiratory volume of less than 70%, are at greater risk (3). Patients with preoperative ventilation problems also have increased risk factors for postoperative problems (12). Those patients who experience prolonged ventilation for more than hours have increased (2, 13). Preexisting chronic obstructive pulmonary disease (COPD) is considered to be a major risk for CABG patients in the postoperative period regarding respiratory morbidity and mortality. However, this risk factor is mainly seen in patients with severe COPD, especially in patients aged 75 years or older (1-4).

## 3. Blood Transfusion and Volume Balance

Issues related to blood transfusion and volume status in the operating room, are among the most important intraoperative factors predicting the occurrence of postoperative respiratory problems (4, 10-15). In one study, it was demonstrated that these factors had a determining role in postoperative pulmonary morbidity and mortality, mainly through their effects on the occurrence of readmission after cardiac surgery with fast-track recovery, in this study, the main reason for ICU readmission was respiratory distress. The study demonstrated that in readmitted patients (11-13, 15) and in patients receiving more than four units of packed cells and fresh frozen plasma, the mortality rate was significantly higher. (15-17). Chest tube drainage of more than 500 mL, is also considered to be one of the main independent risk factors for ICU readmission in fast track CABG (12, 15), possibly because it may increase the chances of packed cell transfusion. The efforts performed by clinicians to reduce blood loss (and hence, the need for transfusion) are among the most important key factors for early tracheal extubation and preventing postoperative respiratory complications (17).

## 4. Anticoagulants and Hemostatic Drugs

Clopidogrel (Plavix) when administered before an operation may increase postoperative bleeding and its complications following CABG. Aprotinin is a high molecular weight, nonspecific serine protease inhibitor which could significantly reduce the amount of blood transfusion required (18, 19), however, critics claim that it might not benefit patients in regard to their clinical outcomes in reducing morbidity or mortality (20-24). On the other hand, a low postoperative dose of aprotinin in patients receiving clopidogrel is safe, and has comparable effects regarding postoperative bleeding complications, as a high dose (25).

## 5. Cardiopulmonary Bypass and Perfusion Status

More than half of a century has passed since the introduction of total CPB for cardiac surgery and nowadays it is used thousands of times each day all around the world (26). One of the main untoward effects of CPB is systemic inflammation, which affects various endothelial beds all over the body. The CPB is composed of a multitude of elements, so due to the great number of resulting interactions between these varying components with the blood components, this creates a significant inflammatory reaction, which is due to humoral and cellular reactions of the immune system, and part of this inflammatory response involves the pulmonary system. The inflammatory effects of CPB on the lungs and the untoward effects of the surgery itself, mandates the use of anti-inflammatory strategies in order to decrease the severity of pulmonary micro-injuries and inflammation (27-29), although many of these anti-inflammatory approaches have not been as successful as expected (28, 29).

It has been demonstrated that cardiopulmonary bypass with longer durations (eg, in one study, surgery took more than 77 minutes), increases the risk for postoperative complications including respiratory complications (3, 15, 30, 31). There are also studies which demonstrate that in patients undergoing CABG without cardiopulmonary bypass (off-pump CABG), that the time needed for tracheal extubation is shorter than on-pump CABG patients (17). This means that earlier tracheal extubation may be performed in patients receiving off-pump CABG. However, this finding has not always been confirmed (1-3, 17, 32-34).

## 6. Preexisting Arrhythmias

Postoperative atrial fibrillation (AF) has been shown to be a risk factor for prediction of post-operative long-term mortality, therefore, using warfarin as an anticoagulant could improve survival (3, 31). The main reason why underlying AF could aggravate the respiratory outcome relies on a number of factors, including; increased pulmonary arterial pressure due to impaired contraction of the left atrium, increased risk for production of atrial clots, especially in the left atrial appendage (LAA), lack of effective contractile force of the LAA and a multitude of other proposed mechanisms (31-33, 35).

## 7. Underlying Function of the Left Ventricle

The underlying functioning of the myocardium is a major predictive factor for postoperative clinical outcomes. It has been shown that patients with preexisting low ejection fraction (EF) are at a higher risk of developing respiratory problems than patients with normal EF (11, 12, 32-36). Impaired left ventricular ejection fraction (LVEF) of less than 30%, history of previous myocardial infarction (36, 37), and preexisting congestive heart failure, age, diabetes mellitus and arterial hypertension, have all been shown to be independent, significant predictors for developing these respiratory problems (30, 31, 35). Low EF especially, has a determining role in patients with a preexisting cardiac problem, mainly valvular heart diseases, such as low gradient aortic stenosis (AS). In such cases, a low EF would be a predictor of postoperative morbidity after aortic valve replacement. This factor affects patient outcomes in such a way that it greatly influences the process of clinical decision making, in order to use or discard surgical methods for treating the diseased valve. Postoperative renal and respiratory complications are among the other important factors in these patients (33, 34, 36). It has also been demonstrated that in patients undergoing CABG combined with valve disease, there is an increased possibility that the patients would require readmission to the ICU. In such patients, post-operative respiratory complications are the most common reasons for readmission (32-34, 36-38). In addition, a history of myocardial infarction during the last 90 days and a previous history of cardiac surgery, are both considered to be important predictors of postoperative respiratory problems (36-40).

## 8. Peripheral Arterial Disease

Peripheral vascular diseases, mainly in the category of peripheral arterial disease, are considered to be one of the main independent risk factors for ICU readmission in fast track CABG. In these patients, underlying peripheral vascular disease has high rates of adverse outcomes (5, 11-13, 37-40).

## 9. Preexisting Endocrine Disorders

Diabetes mellitus is one of the most important endocrine disorders. It is also, considered to be a major risk factor for patients undergoing CABG, which could predict the possibility of postoperative mortality and morbidity in these patients, especially for diabetic patients with respiratory problems (30-38). On the other hand, there is a great amount of current evidence that demonstrates a very clear difference between, well controlled and poorly controlled diabetes mellitus, in predicting respiratory problems, hence, controlling the level of blood glucose in diabetics (at least below 200 mg/dL blood levels) would greatly affect the overall clinical outcome, including respiratory outcomes, in patients undergoing cardiac surgery, especially CABG, compared with poorly controlled diabetics (35-37).

Subclinical hypothyroidism could increase the chances for postoperative complications, including postoperative respiratory complications, however, it is still unproven that preoperative administration thyroxin can prevent this risk factor (41, 42).

## 10. Operating Room Management Factors

The weekly schedule of the operation has been studied as a determinant of outcomes, since elective surgery is usually scheduled for weekdays during ‘normal’ working hours (43-46). Regarding the preoperative risk factors for postoperative pulmonary functions, this factor was not a main determinant of postoperative risk status. The particular time of the surgery; during the day, during the week or during the year, was not related to poor respiratory outcomes in elective CABG (3-5). So, this would not be considered as a risk factor for CABG patients.

On the other hand, the efforts performed by the clinicians to reduce the operation time are among the most important key factors for early tracheal extubation and the prevention of postoperative respiratory complications (16).

## 11. Age

Old age is considered to be an independent risk factor predicting postoperative complications in many studies (2-4, 11-13, 16). Older aged patients (age ≥ 70 years old) is among the most important factors which could impose a greater risk on the CABG patients, as they need to be able to tolerate severely decreased levels of systemic oxygenation and systemic hypoxemia after myocardial revascularization (29, 30). Therefore, clinicians should administer effective treatments for such patients in order to prevent postoperative respiratory problems, of these, pulmonary recruitment strategies (including positive end expiratory pressure or PEEP) need to be mentioned as the top choice of such strategies (1, 29-34, 36-41). In another study, it was demonstrated that one of the main independent risk factors for ICU readmission in fast track CABG, is a patient’s age of more than 65 years (11-13). All of the above findings should be tempered with the fact that the more age increases above 65 years, there is an increased risk for postoperative complications (36-40).

## 12. Ethnic Factors

There are studies that demonstrate ethnic lifestyles may be a strong risk factor for the prediction of postoperative clinical status, including respiratory complications. However, this has not yet been fully elucidated and needs larger confirmatory studies to verify the value of ethnicity regarding perioperative complications (47-49).

### 12.1. Gender

Female patients seem to be at a slightly higher risk of postoperative respiratory complications (1-3, 44-48).

### 12.2. Obesity

Obesity is a major risk factor in predicting the chances of postoperative respiratory failure (3, 12, 13, 15). This effect is both due to the direct effects of obesity on the respiratory system and also, due to the effects of obesity on other organ systems, which could in turn affect the respiratory system (48-52). Although the definite margin for body mass index (BMI) considered as the index of obesity and its role in increasing the risk of respiratory problems has been identified, the effect of this factor is of such importance that even at lower ranges, an increased BMI figure may affect pulmonary outcomes.

### 12.3. History of Smoking

Patients, who are current smokers, are at increased risk for postoperative respiratory problems. This is directly due to the effects of smoking and indirectly due to the effects of smoking on other systems, like the cardiovascular system and the respiratory system, as well as in its role of increasing the risk of pulmonary infections (1, 40, 41, 53). Moreover, a number of smokers also have a history of abuse of other compounds (13). Even though a current and active history of smoking is important, a previous history of smoking, which has not continued up to the time of the operation, should also not be neglected (53).

### 12.4. Preexisting Renal Failure

Increased blood urea nitrogen (BUN) levels or the serum creatinine level in the preoperative period, are independent and potent risk factors, which elevate the chances of postoperative respiratory complications (1-3, 13, 16, 53). Though this is a well-known fact, the exact preoperative levels involved of blood BUN and/or creatinine are still controversial.

### 12.5. Preexisting Arterial Hypertension

Underlying arterial hypertension is considered to be a definitive risk factor, which could predict postoperative pulmonary complications (28, 30).

### 12.6. Condition of the Operation

There are a considerable number of studies which have demonstrated that re-do operations (re-do CABG) and emergency CABG may increase the risk for postoperative pulmonary morbidity and mortality (3, 12, 13, 15). Urgent surgery and emergency surgery are both considered as risk factors for postoperative pulmonary complications. Emergency surgery in particular, is considered to be a risk factor, which is usually associated with an underlying coexisting disease (2, 5).

## 13. Administration of Preoperative Inotropic Agents

Administration of preoperative inotropic agents could increase the risk of postoperative pulmonary morbidity and mortality (3, 11-13, 15).

## 14. Patient Risk Classification Status

These patient classes are at increased risk for postoperative mortality and morbidity (3, 11-13, 54-60):

Higher classes of the New York Heart Association, above class II (3, 12, 13) higher classes of the Canadian Cardiovascular Society, above class II (12, 13); higher classes of the Society for Thoracic Surgeons cardiac risk model, above class II (13), higher classes of the ASA classification (3, 12), and higher classes of European cardiac surgical patients, namely Euroscore (11, 54-56).

## 15. Conclusions

Previous studies have demonstrated that preexisting conditions can affect postoperative clinical outcomes. Therefore, an assessment of the following factors could help clinicians improve the outcomes of their patients.

Preexisting chronic obstructive pulmonary disease, blood transfusion and volume balance, anticoagulant and hemostatic drugs, cardiopulmonary bypass and perfusion status, preexisting arrhythmias, underlying function of the left ventricle, peripheral arterial disease, preexisting endocrine disorders, operating room management factors, age, ethnic factors, female gender, obesity, history of smoking, preexisting renal failure, preexisting arterial hypertension, condition of the operation, administration of preoperative inotropic agents, and patient risk classification status.

## References

[1] Reddy SL, Grayson AD, Griffiths EM, Pullan DM, Rashid A (2007). Logistic risk model for prolonged ventilation after adult cardiac surgery.. Ann Thorac Surg..

[2] Faritous ZS, Aghdaie N, Yazdanian F, Azarfarin R, Dabbagh A (2011). Perioperative risk factors for prolonged mechanical ventilation and tracheostomy in women undergoing coronary artery bypass graft with cardiopulmonary bypass.. Saudi J Anaesth..

[3] Antunes PE, de Oliveira JF, Antunes MJ (2009). Risk-prediction for postoperative major morbidity in coronary surgery.. Eur J Cardiothorac Surg..

[4] Hannan EL, Samadashvili Z, Lahey SJ, Culliford AT, Higgins RS, Jordan D (2010). Predictors of postoperative hematocrit and association of hematocrit with adverse outcomes for coronary artery bypass graft surgery patients with cardiopulmonary bypass.. J Card Surg..

[5] Tan PJ, Xu M, Sessler DI, Bashour CA (2009). Operation timing does not affect outcome after coronary artery bypass graft surgery.. Anesthesiology..

[6] Szeles TF, Yoshinaga EM, Alenca W, Brudniewski M, Ferreira FS, Auler JO (2008). Hypoxemia after myocardial revascularization: analysis of risk factors.. Rev Bras Anestesiol..

[7] Cywinski JB, Xu M, Sessler DI, Mason D, Koch CG (2009). Predictors of prolonged postoperative endotracheal intubation in patients undergoing thoracotomy for lung resection.. J Cardiothorac Vasc Anesth..

[8] Ben-Abraham R, Efrati O, Mishali D, Yulia F, Vardi A, Barzilay Z (2002). Predictors for mortality after prolonged mechanical ventilation after cardiac surgery in children.. J Crit Care..

[9] Kollef MH, Wragge T, Pasque C (1995). Determinants of mortality and multiorgan dysfunction in cardiac surgery patients requiring prolonged mechanical ventilation.. Chest..

[10] Ferasatkish R, Dabbagh A, Alavi M, Mollasadeghi G, Hydarpur E, Moghadam AA (2008). Effect of magnesium sulfate on extubation time and acute pain in coronary artery bypass surgery.. Acta Anaesthesiol Scand..

[11] Branca P, McGaw P, Light R (2001). Factors associated with prolonged mechanical ventilation following coronary artery bypass surgery.. Chest..

[12] Toraman F, Senay S, Gullu U, Karabulut H, Alhan C (2010). Readmission to the intensive care unit after fast-track cardiac surgery: an analysis of risk factors and outcome according to the type of operation.. Heart Surg Forum..

[13] Azarasa M, Azarfarin R, Changizi A, Alizadehasl A (2009). Substance use among Iranian cardiac surgery patients and its effects on short-term outcome.. Anesth Analg..

[14] Dabbagh A, Rajaei S, Shamsolahrar MH (2010). The effect of intravenous magnesium sulfate on acute postoperative bleeding in elective coronary artery bypass surgery.. J Perianesth Nurs..

[15] Mangano DT, Tudor IC, Dietzel C (2006). The risk associated with aprotinin in cardiac surgery.. N Engl J Med..

[16] Mangano DT, Miao Y, Vuylsteke A, Tudor IC, Juneja R, Filipescu D (2007). Investigators of The Multicenter Study of Perioperative Ischemia Research Group; Ischemia Research and Education Foundation. Mortality associated with aprotinin during 5 years following coronary artery bypass graft surgery.. JAMA..

[17] Sato M, Suenaga E, Koga S, Matsuyama S, Kawasaki H, Maki F (2009). Early tracheal extubation after on-pump coronary artery bypass grafting.. Ann Thorac Cardiovasc Surg..

[18] Takagi H, Manabe H, Kawai N, Goto SN, Umemoto T (2009). Aprotinin increases mortality as compared with tranexamic acid in cardiac surgery: a meta-analysis of randomized head-to-head trials.. Interact Cardiovasc Thorac Surg..

[19] Augoustides JG (2008). Perioperative safety of aprotinin in coronary artery bypass graft surgery: is there life after BART?. Drug Saf..

[20] Henry D, Carless P, Fergusson D, Laupacis A (2009). The safety of aprotinin and lysine-derived antifibrinolytic drugs in cardiac surgery: a meta-analysis.. CMAJ..

[21] Ide M, Bolliger D, Taketomi T, Tanaka KA (2010). Lessons from the aprotinin saga: current perspective on antifibrinolytic therapy in cardiac surgery.. J Anesth..

[22] Hill GE, Alonso A, Spurzem JR, Stammers AH, Robbins RA (1995). Aprotinin and methylprednisolone equally blunt cardiopulmonary bypass-induced inflammation in humans.. J Thorac Cardiovasc Surg..

[23] Ovrum E, Tangen G, Tollofsrud S, Ringdal MA, Oystese R, Istad R (2010). Low postoperative dose of aprotinin reduces bleeding and is safe in patients receiving clopidogrel before coronary artery bypass surgery. A prospective randomized study.. Interact Cardiovasc Thorac Surg..

[24] El-Chami MF, Kilgo P, Thourani V, Lattouf OM, Delurgio DB, Guyton RA (2010). New-onset atrial fibrillation predicts long-term mortality after coronary artery bypass graft.. J Am Coll Cardiol..

[25] Basiladze L, Prangishvili A, Chapidze G, Pirvelashvili E, Bakhutashvili Z (2009). Coronary artery bypass grafting in patients with low ejection fraction.. Georgian Med News..

[26] Paparella D, Yau TM, Young E (2002). Cardiopulmonary bypass induced inflammation: pathophysiology and treatment. An update.. Eur J Cardiothorac Surg..

[27] Kunes P, Mandak J, Harrer J, Kolackova M, Andrys C, Holicka M (2010). Up-regulation of the Apo/Fas (CD95) complex on neutrophils harvested during cardiac surgery: distinct findings in patients operated on with or without the use of cardiopulmonary bypass.. Perfusion..

[28] Warren OJ, Smith AJ, Alexiou C, Rogers PL, Jawad N, Vincent C (2009). The inflammatory response to cardiopulmonary bypass: part 1--mechanisms of pathogenesis.. J Cardiothorac Vasc Anesth..

[29] Warren OJ, Watret AL, de Wit KL, Alexiou C, Vincent C, Darzi AW (2009). The inflammatory response to cardiopulmonary bypass: part 2--anti-inflammatory therapeutic strategies.. J Cardiothorac Vasc Anesth..

[30] Mistiaen W, Van Cauwelaert P, Muylaert P, De Worm E (2009). A thousand pericardial valves in aortic position: risk factors for postoperative acute renal function impairment in elderly.. J Cardiovasc Surg (Torino)..

[31] Chikwe J, Croft LB, Goldstone AB, Castillo JG, Rahmanian PB, Adams DH (2009). Comparison of the results of aortic valve replacement with or without concomitant coronary artery bypass grafting in patients with left ventricular ejection fraction < or =30% versus patients with ejection fraction >30%.. Am J Cardiol..

[32] Ahmed WA, Tully PJ, Baker RA, Knight JL (2009). Survival after isolated coronary artery bypass grafting in patients with severe left ventricular dysfunction.. Ann Thorac Surg..

[33] El Diasty M, Gonzalez JA, Perez J, Cid F, Mosquera V, Cuenca J (2009). Early results of off-pump coronary artery bypass graft surgery using bilateral internal thoracic artery grafts in octogenarian patients during ten years.. Interact Cardiovasc Thorac Surg..

[34] Cooper WA, Thourani VH, Guyton RA, Kilgo P, Lattouf OM, Chen EP (2009). Racial disparity persists after on-pump and off-pump coronary artery bypass grafting.. Circulation..

[35] Cislaghi F, Condemi AM, Corona A (2009). Predictors of prolonged mechanical ventilation in a cohort of 5123 cardiac surgical patients.. Eur J Anaesthesiol..

[36] Cwynar R, Albert NM, Butler R, Hall C (2009). Factors associated with long hospital length of stay in patients receiving warfarin after cardiac surgery.. J Cardiovasc Nurs..

[37] Luciani N, Nasso G, Gaudino M, Abbate A, Glieca F, Alessandrini F (2003). Coronary artery bypass grafting in type II diabetic patients: a comparison between insulin-dependent and non-insulin-dependent patients at short- and mid-term follow-up.. Ann Thorac Surg..

[38] Kor DJ, Warner DO, Alsara A, Fernandez-Perez ER, Malinchoc M, Kashyap R (2011). Derivation and diagnostic accuracy of the surgical lung injury prediction model.. Anesthesiology..

[39] Fukui T, Shimokawa T, Manabe S, Morita S, Takanashi S (2009). Prior inferior myocardial infarction has worse early outcomes in patients undergoing coronary artery bypass grafting than prior anterior myocardial infarction.. Ann Thorac Surg..

[40] Litmathe J, Kurt M, Feindt P, Gams E, Boeken U (2009). Predictors and outcome of ICU readmission after cardiac surgery.. Thorac Cardiovasc Surg..

[41] O’Rourke DJ, Quinton HB, Piper W, Hernandez F, Morton J, Hettleman B (2004). Survival in patients with peripheral vascular disease after percutaneous coronary intervention and coronary artery bypass graft surgery.. Ann Thorac Surg..

[42] Park YJ, Yoon JW, Kim KI, Lee YJ, Kim KW, Choi SH (2009). Subclinical hypothyroidism might increase the risk of transient atrial fibrillation after coronary artery bypass grafting.. Ann Thorac Surg..

[43] Sirvinskas E, Andrejaitiene J, Raliene L, Nasvytis L, Karbonskiene A, Pilvinis V (2008). Cardiopulmonary bypass management and acute renal failure: risk factors and prognosis.. Perfusion..

[44] Weiss ES, Chang DD, Joyce DL, Nwakanma LU, Yuh DD (2008). Optimal timing of coronary artery bypass after acute myocardial infarction: a review of California discharge data.. J Thorac Cardiovasc Surg..

[45] Papadimos TJ, Habib RH, Zacharias A, Schwann TA, Riordan CJ, Durham SJ (2005). Early efficacy of CABG care delivery in a low procedure-volume community hospital: operative and midterm results.. BMC Surg..

[46] Elahi M, Matata B, Yii M (2008). Ethnicity and adverse operative outcomes among Australian patients undergoing first-time isolated coronary artery bypass graft surgery.. Int Surg..

[47] Sawatzky JA, Naimark BJ (2009). The coronary artery bypass graft surgery trajectory: Gender differences revisited.. Eur J Cardiovasc Nurs..

[48] Leeper B (2009). Impact of obesity on care of postoperative coronary bypass patients.. Crit Care Nurs Clin North Am..

[49] Pan W, Hindler K, Lee VV, Vaughn WK, Collard CD (2006). Obesity in diabetic patients undergoing coronary artery bypass graft surgery is associated with increased postoperative morbidity.. Anesthesiology..

[50] Tolpin DA, Collard CD, Lee VV, Elayda MA, Pan W (2009). Obesity is associated with increased morbidity after coronary artery bypass graft surgery in patients with renal insufficiency.. J Thorac Cardiovasc Surg..

[51] Virani SS, Nambi V, Lee VV, Elayda MA, Pan W, Petersen LA (2009). Obesity: an independent predictor of in-hospital postoperative renal insufficiency among patients undergoing cardiac surgery?. Tex Heart Inst J..

[52] Kulik A, Ruel M (2009). Statins and coronary artery bypass graft surgery: preoperative and postoperative efficacy and safety.. Expert Opin Drug Saf..

[53] Kinlin LM, Kirchner C, Zhang H, Daley J, Fisman DN (2010). Derivation and validation of a clinical prediction rule for nosocomial pneumonia after coronary artery bypass graft surgery.. Clin Infect Dis..

[54] Rady MY, Ryan T (1999). Perioperative predictors of extubation failure and the effect on clinical outcome after cardiac surgery.. Crit Care Med..

[55] Canver CC, Chanda J (2003). Intraoperative and postoperative risk factors for respiratory failure after coronary bypass.. Ann Thorac Surg..

[56] Spivack SD, Shinozaki T, Albertini JJ, Deane R (1996). Preoperative prediction of postoperative respiratory outcome. Coronary artery bypass grafting.. Chest..

[57] Nashef SA, Roques F, Hammill BG, Peterson ED, Michel P, Grover FL (2002). Validation of European System for Cardiac Operative Risk Evaluation (EuroSCORE) in North American cardiac surgery.. Eur J Cardiothorac Surg..

[58] Roques F, Michel P, Goldstone AR, Nashef SA (2003). The logistic EuroSCORE.. Eur Heart J..

[59] Choong CK, Sergeant P, Nashef SA, Smith JA, Bridgewater B (2009). The EuroSCORE risk stratification system in the current era: how accurate is it and what should be done if it is inaccurate?. Eur J Cardiothorac Surg..

[60] Nashef SA (2009). Editorial comment: What to do with EuroSCORE in 2009?. Eur J Cardiothorac Surg..

